# An assessment of the diagnostic criteria for sessile serrated adenoma/polyps: SSA/Ps using image processing software analysis for Ki67 immunohistochemistry

**DOI:** 10.1186/1746-1596-7-59

**Published:** 2012-05-29

**Authors:** Yukari Fujimori, Takahiro Fujimori, Johji Imura, Tamotsu Sugai, Takashi Yao, Ryo Wada, Yoichi Ajioka, Yasuo Ohkura

**Affiliations:** 1 Department of Surgical and Molecular Pathology, Dokkyo Medical University School of Medicine, 880 Kitakobayashi, Mibu, Shimotsuga, Tochigi, 321-0293, Japan; 2 Department of Diagnostic Pathology, Graduate School of Medicine and Pharmaceutical Sciences, University of Toyama, 2630 Sugitani, Toyama, 930-0194, Japan; 3 Division of Diagnostic Molecular Pathology, Department of Pathology, School of Medicine, Iwate Medical University, 19-1, Morioka City, 020-8505, Japan; 4 Department of Human Pathology, Juntendo University School of Medicine, 113-8421, Tokyo, Japan; 5 Department of Pathology, Juntendo Shizuoka Hospital of Juntendo University School of Medicine, Shizuoka, Japan; 6 Division of Molecular and Diagnostic Pathology, Niigata University Graduate School of Medical and Dental Sciences, Niigata, 951-8510, Japan; 7 Department of Pathology, Kyorin University School of Medicine, 6-20-2 Shinkawa, Mitaka, Tokyo, 181-8611, Japan

**Keywords:** Sessile serrated adenoma/ polyp, Ki-67, Immunohistochemistry, Image processing software, Morphologic characteristics

## Abstract

**Background:**

Serrated polyps belong to a heterogeneous group of lesions that are generally characterized morphologically. This type of lesion is thought to be the precursor of sporadic carcinomas with microsatellite instability, and probably also the precursor for CpG island-methylated microsatellite-stable carcinomas. For practical purposes, according to the 2010 WHO classification, the diagnostic criteria for sessile serrated adenomas/polyps (SSA/Ps) was established by the research project “Potential of Cancerization of Colorectal Serrated Lesions” led by the Japanese Society for Cancer of the Colon and Rectum. The aim of this study was to evaluate the validity of the morphologic characteristics established in Japan by using immunohistochemical staining for Ki-67.

**Methods:**

To calculate the target cells, 2 contiguous crypts which could be detected from the bottom of the crypt to the surface of the colorectal epithelium were selected. To validate the proliferative activity, we evaluated the percentage and the asymmetrical staining pattern of Ki67 positive cells in each individual crypt. To examine the immunoreactivity of Ki67, computer-assisted cytometrical analysis was performed.

**Results:**

SSA/Ps had a higher proliferative activity as compared to hyperplastic polyps (HPs) based on the difference in incidence of Ki67 positive cells, and the former also exhibited a significantly higher asymmetric distribution of these cells as compared to HPs, even in lesions with a diameter <10 mm.

**Conclusion:**

We conclude that assessment of the pathological findings of SSA/Ps, including crypt dilation, irregularly branching crypts, and horizontally arranged basal crypts (inverted T- and/or L-shaped crypts) is appropriate to show a significantly higher proliferative activity as compared to HPs. Further, the use of two-dimensional image analysis software is an objective and reproducible method for this type of histological examination.

**Virtual slides:**

The virtual slides for this article can be found here: http://www.diagnosticpathology.diagnomx.eu/vs/6718091416698112

## Background

The new concept of the serrated pathway was proposed by Snover et al., although it is assumed that all colorectal carcinomas arise from sporadic adenomas (the Fearon-Vogelstein model) or *de-novo* carcinogenesis [1]. Serrated polyps belong to a heterogeneous group of lesions that are classified according to their morphology/morphological phenotypes. These types of lesions are thought to be the precursor of sporadic carcinomas with microsatellite instability (MSI) and are probably also the precursor for CpG island-methylated microsatellite-stable carcinomas [2-4]. In 1996, a report by Torlakovic and Snover et al. showed lesions that bore some resemblance to hyperplastic polyps (HPs), which were found in association with the development of adenocarcinoma [5]. In 2003, a subsequent report demonstrated that these lesions, which had been mistaken for HPs, were found to account for 18% of all serrated polyps. They designated these lesions as “sessile serrated adenomas/polyps” (SSA/Ps) to distinguish them from conventional HPs [6]. In 2010, the morphology of SSA/Ps, characterized by a serrated architecture of the epithelial compartment, was first presented in the fourth edition of the WHO Classification of Tumors of the Digestive System [7]. For practical purposes, according to the WHO classification, the diagnostic criteria for SSA/Ps was established by the research project “Potential of Cancerization of Colorectal Serrated Lesions” led by the Japanese Society for Cancer of the Colon and Rectum (Yao T, et al) [8]. The aim of the current study was to evaluate the validity of the morphologic characteristics established in Japan using immunohistochemical staining for Ki-67.

## Methods

### Tissue samples and histology

Two hundred and seventy-nine specimens from 223 patients were derived from the Department of Pathology archives of Dokkyo Medical University between July 2008 and December 2010. Sixty cases obtained by biopsy, 90 cases by colonoscopic polypectomy, and 88 cases by endoscopic resection were included in the initial assessment. The fourth edition of the WHO Classification of Tumors of the Digestive System was used to distinguish HPs and SSA/Ps from other polyps. As described above, SSA/Ps were distinguished from conventional HPs on the basis of the following features: 1) crypt dilation, 2) irregularly branching crypts, and 3) horizontally arranged basal area of the crypts (inverted T- and/or L-shaped crypts). The serrated lesions which had more than 2 of these findings were diagnosed as SSA/Ps, and those with only one of these findings were designated as intermediate type. Others that contained none of these features were regarded as HPs. Because of sampling issues or poor orientation of the specimen, some of the polyps were excluded. Finally, 68 cases were used for the comparison. The authors and a pathologist (T.F.) examined all samples.Hematoxylin and eosin staining was performed as usual.Hematoxylin and eosin-stained sections of each sample were employed for pathological examinations.

### Immunohistochemical staining for Ki67

Immunohistochemical staining for Ki67 was performed with a LSAB-2 kit (LSAB2 System-HRP; DAKO, Carpinteria, CA, USA) as described previously [9,10]. The 4 μm thick sections were placed on slides, deparaffinized, and dehydrated. They were then placed in 0.01 M citrate buffer (pH 6.0) and treated by microwave heating (400 W, 95°C; MI-77; Azumaya, Tokyo, Japan) for 40 minutes to facilitate antigen retrieval. Then, the sections were pretreated with 0.3% H_2_O_2_ in methanol at room temperature to quench endogenous peroxidase activity. This was followed by blocking with Protein Block Serum-Free (Dako, USA) for 30 minutes, and incubation with anti-Ki-67 antibody (1:50 clone MIB-1; Dako, Japan) for 1 hour. Thereafter, the sections were incubated with biotinylated secondary antibody for 15 minutes, washed with PBS, and treated with peroxidase-conjugated streptavidin for 20 min. Finally, the sections were visualized by incubating in 3, 3′-diaminobenzidine tetrahydrochloride with 0.05% H_2_O_2_ (Liquid DAB + Substrate Chromogen System; Dako, USA) for 3 min and then counterstained with Carazzi’s hematoxylin.

### Evaluation of immunohistochemical Ki67 expression

To assess the target cells, 2 contiguous crypts which could be visualized from the bottom of the crypt to the surface of the colorectal epithelium were selected. Immunostained sections were evaluated by 3 of the authors and a pathologist, and Ki-67 positivity was evaluated as described previously. In our first evaluation, the proportion of Ki-67 positive cells was calculated as the percentage of positive cells in 2 crypts. In the second step, to evaluate the asymmetrical staining pattern of Ki-67 positive cells in each individual crypt, the distance between the immunopositive cells located closest to the surface and the bottom of the crypt was measured. Computer-assisted cytometrical analysis of Ki-67 immunoreactivity was performed using the WinROOF image processing software (Mitani Corp., Tokyo, Japan) [11].

### Statistical analysis

The proportion of Ki-67 positively-stained cells and the asymmetrical location of proliferating cells on either side of the crypts were analyzed by the Mann–Whitney *U*-test. Continuous variables were expressed as mean ± SD or, where indicated, as median and interquartile range (IQR). A P-value < 0.05 was considered significant.

## Results

### Clinical characteristics

**Figure 1:**
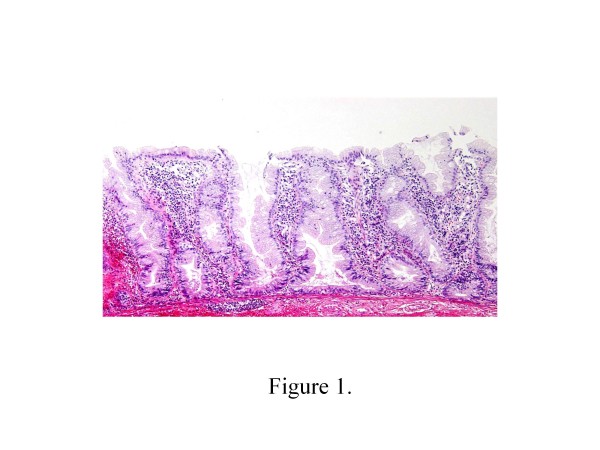
**Characteristic histological features of SSA/P(Hematoxylin and eosin staining).** The crypts showed dilation, serration, irreguraly branching and horizontally arranging (L-shaped, inverted T-shaped or anchor shaped) at the base.

Histological examination of the Hematoxylin and eosin-stained specimens of the 68 serrated polyps without cytological dysplasia showed that 14 of them were HPs and 24 were SSA/Ps (Figure 1), while 30 were of the intermediate type. Clinicopathologic characteristics of SSA/P, HP, and intermediate type polyps are shown in Table 1. The percentage of females with SSA/Ps was higher than that with HPs. The mean size of SSA/Ps (mean 9 mm; range 7–15 mm) was larger than that of HPs (mean 5 mm; range 2–8 mm). Forty-two percent of SSA/Ps was over 10 mm in size. The percentage of SSA/Ps on the right side of the colon was more frequent than that of HPs. Macroscopic flat-elevated findings tended to be higher in SSA/Ps compared with HPs. Further, the mean size of the intermediate type polyps (mean 7 mm; range 5–10 mm) was larger than that of HPs but smaller than that of SSA/Ps. There were no statistically significant differences in age distribution and location between the intermediate type and the other two types (Table 2).

**Table 1:** Diagnostic criteria of SSA/P from Project Research “Potential of Cancerization of Colorectal Serrated Lesions” of Japanese Society for Cancer of the Colon and Rectum

SSAP/P is composed of serrated cryptal epithelium with aberrant compartmentalization, essentially characterized by the architectural abnormalities listed below	
If the serrated lesion have more than 2 findings of them, it can be diagnosed as SSA/P	
^(1)^	Crypt dilation
^(2)^	Irregularly branching crypts
^(3)^	Horizontally arranged basal crypts (Inverted T- and/or L-shaped crypts)

**Table 2:** Clinicopathologic characteristics of SSA/P, HP and Intermediated type

	HP	SSAP	*p*^a^	intermediate
	(n=14)	(n=24)	(SSAP/P *vs*. HP)	(n=30)
Sex: Male/Female	12/2	13/11	.03394	21/9
Age (y), median (range)	52.5 (46–64)	62.5 (51.5–73)	.00086	55 (51–63)
Size (mm), median (range)	5 (2–8)	9 (7–15)		7 (5–10)
Location (%)				
Right colon	3 (21)	17 (71)		15 (50)
Left colon	5 (36)	6 (25)	.00209	10 (33)
Rectum	6 (43)	1 (4)		5 (17)
Endoscopic morphology				
Ip	1 (7)	0 (0)		0 (0)
non-Ip	9 (64)	22 (92)	<.001	24 (80)
UN	4 (29)	2 (8)		6 (20)

^a^ Mann-Whitney *U* test or ×2 test.

HP: Hyperplastic polyp.

SSA/P: Sessile serrated adenoma/polyp.

### Immunohistochemical Ki-67 expression in SSA/Ps and HPs

**Figure 2:**
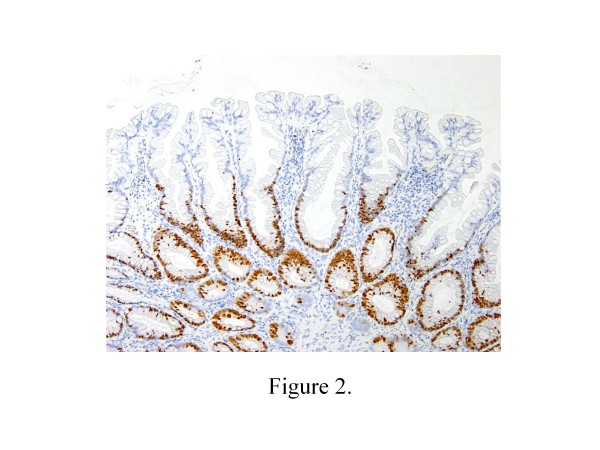
**Immunohistochemical findings for Ki-67 in HP and SSA/P.** Ki-67 positive cells were seen in the bottom and surface of individual crypt of HP (Figure 2) and SSA/P (Figure 3).

**Figure 3:**
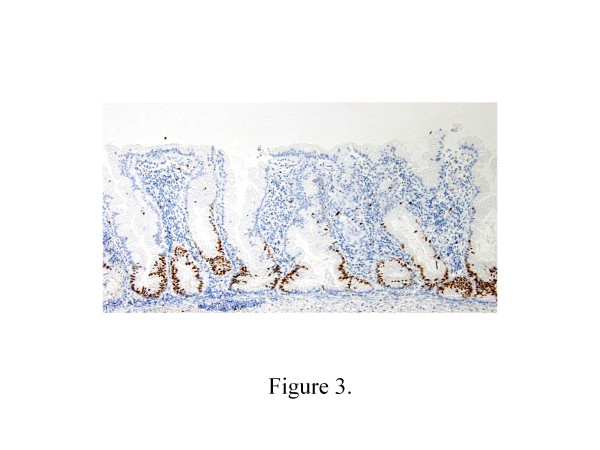
**Immunohistochemical findings for Ki-67 in HP and SSA/P.** Ki-67 positive cells were seen in the bottom and surface of individual crypt of HP (Figure 2) and SSA/P (Figure 3).

The percentage of Ki-67 positive cells in the SSA/Ps (mean 43.4%, range 35.5–50.3%) (Figure 2) was significantly higher than that in the HPs (mean 30.9%, range 22.8–34.5%) (*p* = 0.00019) (Figure 3). There was also a statistically significant difference in the distribution of Ki-67 positive cells between SSA/Ps (mean 12.9%, range 8.7–18.1%) and HPs (mean 8.3%, range 6.9–10.4%) (*p* = 0.00562) (Table 3).

**Table 3:** Immunohistochemical Ki67 expression in SSA/P and HP

	HP (n=14)	SSA/P (n=24)	*p*^a^
Ki67 positivity* (%) 19	30.9 (22.8–34.5)	43.4 (35.5–50.3)	.000
Asymmetry of the location** (%) 62	8.3 (6.9–10.4)	12.9 (8.7–18.1)	.005

^a^ Mann-Whitney *U* test.

* Frequency of Ki67 positive cells.

** Asymmetry of the location of Ki67 positive cells in each individual crypt.

In addition, we evaluated the percentage and the asymmetrical staining pattern of the Ki-67 positive cells in each individual crypt in lesions with a diameter <10 mm. For this purpose, fourteen cases of SSA/Ps and 12 cases of HPs were selected. The percentage of Ki-67 positive cells in lesions that were less than 10 mm in the SSA/P cases (mean 41.75%, range 34.5–45.8%) were significantly higher than in the HP specimens (30.9%, 24.25–35.75%) (*p* = 0.00692). Further, the distribution of Ki-67 cells in SSA/Ps that were less than 10 mm (11.65%, 8.3–17.9%) was more asymmetrical than in HPs (8.65%, 5.2–10.7%) (*p* = 0.03075) (Table 4), a difference that was statistically significant.

**Table 4:** Immunohistochemical Ki67 expression in SSA/P and HP (<10mm)

	HP (n=12)	SSA/P (n=14)	*p*^a^
Ki67 positivity* (%) .00692	30.9 (24.25–35.75)	41.75 (34.5–45.8)	
Asymmetry of the location** (%) .03075	8.65 (5.2–10.7)	11.65 (8.3–17.9)	

^a^ Mann-Whitney *U* test.

* Frequency of Ki67 positive cells.

** Asymmetry of the location of Ki67 positive cells in each individual crypt.

Interestingly, in the intermediate type, the mean percentage of the positive cells was 35.85% (range 31.05–45.35%), and the asymmetrical distribution of the Ki-67 positive cells was 11.65% (range 8.3–17.9%), values which were intermediate between SSA/Ps and HPs.

## Discussion

In our previous report, proliferative activity of the colon epithelium was compared between SSA/Ps and HPs using Ki-67 immunostaining [12]. Our findings showed that the proportion of Ki-67 positive cells was higher in SSA/Ps as compared to HPs. A difference in the distribution pattern of the proliferative compartment revealed that Ki-67 immunostaining is useful to differentiate between SSA/Ps and HPs. However, the number of cells was visually counted, resulting in lack of objectivity and reproducibility of measurement. In this study, although we strived to circumvent the previous issues by counting the number of cells in microscopic images (x10) processed by Photoshop Elements 9 using a two-dimensional image analysis software [11], several problems still occurred. The targeted area of the colon mucosa was traced and defined, followed by a count of the number of cells based on color recognition of the cells. In order to maximally differentiate the target cells from the adjacent ones, the margins of the nuclei were identified by enhanced contrast for RGB separation. Although G (green) image was the easiest to assess, maximal and accurate differentiation of the targeted cells from the adjacent cells was still not possible using just the color recognition method. The measurement was, therefore, done with the “Separate Circular Figure” function using the 2D Image Analysis Software, Win-ROOF, by Mitani Corporation, Japan. The maximum and minimum diameters of the cells, the number of candidacy center points, edge threshold, and contrast were set to mark the cell border and in turn, visualize the cells as donut-shaped. Thus, the number of cells could be more accurately counted. However, due to the complicated adjustments of these settings, manual counting function of the image analysis software was utilized, and the number of cells was counted with a touch pen by introducing a liquid crystal touch panel. Moreover, the distance from the base of the crypts to the Ki-67 positive cells on the surface was also defined by a line drawn with a touch pen and automatically measured. These measurements could be automatically transferred to Microsoft Excel and were also useful for data processing. The above-mentioned measurements were performed as a pilot study, which was followed by commencement of the main study.

The protein antigen Ki-67 has been widely used as an indicator of proliferation due to its restricted expression between the S and the M phases of the cell cycle in differentiating cells. In this study, it was confirmed that SSA/Ps had a higher proliferative activity as compared to HPs as shown by the difference in incidence of Ki-67 positive cells. Furthermore, based on the assumption that the proliferative compartment might be an indicator of distribution range, the distance from the base of the crypt to the Ki-67 positive cells on the surface was measured, and it was found that SSA/Ps exhibited a significantly more asymmetric distribution compared with HPs [13]. Moreover, it was revealed that SSA/Ps had a higher proliferative activity with more asymmetric distribution, as demonstrated by proportion of Ki-67 positive cells and distance in a single crypt, respectively, as compared to HPs even in lesions with diameter <10 mm. These results demonstrated that, according to the diagnostic criteria provided in Table 1, in SSA/Ps, even lesions with a diameter < 10 mm had a higher proliferative activity as compared to similar sized lesions in HPs. Thus, we verified here that the common features of SSA/Ps including crypt dilation, irregularly branching crypts, and horizontally arranged basal crypts (inverted T- and/ or L-shaped crypts) are indeed appropriate histological findings to show “none dysplastic, but have an extended proliferative zone [14,15]” as indicated by Riddell. In this study, the intermediate group showed intermediate characteristics between HP and SSA/P, and this may be because this type of lesion is a transitional stage from HP to SSA/P. However, further investigation is required in order to support this finding.

## Conclusion

The pathological characters and the diagnostic criteria for SSA/P was established by the research project “Potential of Cancerization of Colorectal Serrated Lesions” led by the Japanese Society for Cancer of the Colon and Rectum (Y T, et al). As a result of this study, the pathological features of SSA/P including crypt dilation, irregularly branching crypts, and horizontally arranged basal crypts (inverted T- and/ or L-shaped crypts) were found to be appropriate histological findings to show a significantly higher proliferative activity as compared to HP.

## Abbreviations

HP: Hyperplastic polyp; SSA/P: Sessile serrated adenoma/polyp.

## Competing interests

No financial and non-financial competing interests to declare in relation to this manuscript.

## Authors’ contributions

YF was involved in the design of the study and immunohistochemical analysis, and drafted the manuscript. TF and JI conceived the study, was involved in the design and immunohistochemical analysis, and edited the manuscript for intellectual content. TS, TY, RW, YA and YO were involved in the design of the study and pathological diagnosis. All authors read and approved the final manuscript.
